# Characterising and Comparing the Sleep Characteristics and Behaviours of Female and Male Soccer Players: A Cross-Sectional Survey of an Elite Soccer Club

**DOI:** 10.3390/sports13060189

**Published:** 2025-06-19

**Authors:** Nicole Sanders, Rebecca K. Randell, Craig Thomas, Stephen J. Bailey, Tom Clifford

**Affiliations:** 1 School of Sport, Exercise and Health Sciences, Loughborough University, Loughborough LE11 3HT, UK; n.sanders@lboro.ac.uk (N.S.); rebecca.randell@pepsico.com (R.K.R.); s.bailey2@lboro.ac.uk (S.J.B.); 2 Gatorade Sports Science Institute, Life Sciences R&D, PepsiCo, Leicester LE4 1ET, UK; 3 The Northumbria Centre for Sleep Research, Northumbria University, Newcastle NE1 8ST, UK; craigj.thomas@yahoo.com

**Keywords:** sleep hygiene, sport, football

## Abstract

The aim of this cross-sectional study was to evaluate the sleep characteristics and behaviours of senior male, senior female, and under 21 (U21) male elite soccer players using athlete-specific questionnaires. During the preseason/early season period, 74 players from the English Premier League (n = 26, age 26 ± 5 y), Women’s Super League (n = 22, age 25 ± 5 y), and English Premier League 2 (n = 26, age 19 ± 1 y) completed the validated Athlete Sleep Screening Questionnaire (ASSQ) to obtain a sleep difficulty score (SDS) and the Athlete Sleep Behaviour Questionnaire (ASBQ) to obtain a global score and individual behaviours. We found that sleep difficulty scores were higher in senior females (5.9 ± 1.9) than senior males (4.1 ± 1.7) and U21 males (4.3 ± 1.2) (*p* ≤ 0.006), but no severe clinical problems were noted. Global sleep behaviour scores from the ASBQ were worse in senior males (37.9 ± 6.5) and senior females (40.6 ± 7.1) than male U21 players (33.6 ± 4.7) (*p* ≤ 0.021). Senior players consumed more alcohol and stimulants and felt that travel disrupted sleep; females went to bed thirstier, woke more for the bathroom, and ruminated more prior to sleep (all *p* < 0.05). In conclusion, senior female players reported more sleep difficulties than male senior and male U21 players. Behaviours such as pre-bed rumination, nutrition, and travel plans could be targeted to improve sleep quality in soccer players. Study limitations include data drawn from a single club in the off-season.

## 1. Introduction

Athletes and coaches view sleep as an important aspect of recovery [1,2]. Indeed, sleep is thought to play a restorative role in cognitive, psychological, and physiological functioning, meaning poor sleep could negatively influence recovery and performance [3,4,5]. Several studies suggest that poor sleep affects performance in elite athletes; for example, Knufinke-Meyfroyt and colleagues [6] reported slower reaction times after reduced sleep, and Craven et al. [7] found that acute sleep loss compromised exercise performance in activities with a skill element. While cognitive processes appear most impaired by poor sleep [3], reduced skeletal muscle protein synthesis, disturbed endocrine [8] and immune [9] function, and an increased risk of injury [10] are all potentially deleterious consequences of poor sleep.

Poor sleep quality and reduced sleep quantity are often reported by athletes, including soccer players [11]. Despite National Recommendations of at least 7 h sleep per night [12], some studies have shown that soccer players do not achieve this, as measured by wristwatch actigraphy [13,14,15] or subjectively with surveys [16]. Difficulty sleeping is common after soccer matches [14,17] and with travel [15,16,18]. These difficulties may be due to the demanding psychological conditions and high arousal associated with match play [19], bright light exposure [20], high training loads, including overtraining [21,22], or fatigue associated with jet lag from overseas travel. However, there is limited data exploring sleep difficulties outside of matches and long-haul travel in soccer players or the possible behaviours affecting habitual sleep. In particular, there are no studies describing sleep habits and behaviours in elite soccer players competing in the top English leagues, which means there is a lack of knowledge on problematic behaviours that could be targeted with dedicated interventions to support sleep quality.

Some recent studies have assessed whether biological sex mediates sleep quality in elite athletes. One study [23] found that female Japanese collegiate soccer players slept less, and rated sleep quality lower, than male players. Similarly, another [24] reported better sleep quality and quantity in elite male vs. female rugby union players. However, Randell et al. [25] reported no differences in self-reported sleep quality between male and female athletes competing in a range of sports. Recent research suggests that distinct lifestyle factors may influence sleep difficulty in males versus females [26]. For example, Kawasaki and colleagues [26] found that in males, sleep apnoea and turning the heating on were associated with poor subjective sleep quality, whilst in females, alcohol use and noise had a more negative influence. Some females may also encounter sleep difficulty due to the menstrual cycle and resulting hormonal fluctuations or sleep disruption with dysmenorrhea pain [27]. Lower sleep quality has been reported during early follicular and late luteal phases [28] and lower sleep efficiency in the late luteal phase [29], albeit research in this area is limited and equivocal [30,31]. The limited research to date suggests that different behaviours may modulate sleep quality in male and female soccer players; thus, more research is needed to compare their sleep behaviours, enabling more targeted interventions to be developed.

Evidence of age affecting sleep quality between athletes is scarce, but an age-related decline in sleep variables has been observed in young [32] and old [33] non-athletic populations. One study [34] observed that age and total sleep time were inversely related in young female elite gymnasts. While another study [35] found that ~19-year-old male academy soccer players slept over 8 h per night, there are reports that senior male soccer players sleep less than 7 h per night [14,15,16,36]. Differences in sleep difficulties between senior and younger players could be due to a range of lifestyle factors. For example, younger players may also have academic commitments; combining sport and academic commitments was shown to increase sleep variability in student athletes compared to non-athlete student controls [37]. Other research suggests that athletes face distinct physical and psychological stressors at different stages of their careers [38]. Indeed, in young soccer players, one study [39] observed more stress placed on making errors, team performance, and selection in middle adolescents (15–18 years) compared to younger players (12–14 years). Senior athletes may be better equipped to cope with stressors, as mental toughness increases with age [40], but may face more family stressors, such as young children, which can disrupt sleep [41]. The higher physical outputs (sprinting distance and high-intensity burst distance) of senior players versus U18 and U21 players [38], and the increased number of fixtures, may also mediate differences in sleep quantity and quality between senior and younger players. However, there is currently a lack of qualitative and quantitative data comparing the sleep habits of younger and senior soccer players, and, therefore, information on how sleep hygiene strategies may need to be adjusted to target specific age groups is lacking.

Wristwatch actigraphy is commonly used to measure sleep quality in athletes [42,43] and soccer players [14,15,16,44,45]. While actigraphy can identify objective changes in sleep duration and quality, it does not capture any contextual information on sleep. Alternatively, specially designed surveys can capture athletes’ perception of sleep quality, as well as identify any behaviours linked to poor sleep. Although the accuracy of surveys in determining individual sleep metrics is limited, surveys offer a less intrusive means to understand sleep in athletes compared to actigraphy and are especially useful for capturing sleep habits of large groups (e.g., teams and squads) over longer periods of time (e.g., >1 month). As such, two surveys have been developed and validated to assess athletes’ sleep habits: the Athlete Sleep Screening Questionnaire (ASSQ) and the Athlete Sleep Behaviour Questionnaire (ASBQ). Both questionnaires were developed to gather contextual information that can identify poor sleep behaviours on which to base interventions.

Therefore, the aims of this study were to (1) describe the sleep characteristics and difficulties of elite players from the same club in the pre/early season period using the ASSQ and ASBQ and (2) compare any differences between elite senior male, senior female, and U21 male soccer players. This is the first study to describe and compare the sleep characteristics in these elite populations using these surveys, and we hope this study will provide novel information for practitioners to utilise in developing strategies to enhance sleep quality in elite soccer players. We hypothesised that minor sleep difficulties and poor sleep behaviours would be exhibited across all groups and that there would be differences in sleep difficulty between male U21 and male and female senior players.

## 2. Materials and Methods

### 2.1. Participants

This was a cross-sectional, observational study at a professional soccer club. Purposeful sampling was used to recruit elite soccer players in England. To be eligible for the study, volunteers had to be elite soccer players contracted to the same English soccer club in which the research was conducted and playing for either the men’s or women’s first team squads or the male U21 squad. There were no specific exclusion criteria, but all players had to be ≥18 years old. None of the players had a known sleep disorder or other medical condition that could influence the results. In total, 74 male and female soccer players competing in the male English Premier League (EPL), male U21 Premier League 2 Division 1 (PL2), and Women’s Super League (WSL) provided informed consent to take part in this study. The male U21 players would be considered Tier 4, Elite/International Level, and the male and female seniors, Tier 5, World Class, in the Participant Classification framework [46]. Of these, 52 were male; 26 were in the senior squad (age 26 ± 5 y) and 26 were in the under 21 squad (age 19 ± 1 y), and 22 were in the women’s senior squad (age 25 ± 5 y). At the time of questionnaire completion, 2 players were injured and not involved in regular team training. Ethical approval was granted by the Loughborough University Ethics Committee.

An a priori power analysis was performed for a one-way ANOVA using G * Power (Version 3.1.9.6) [47]. A large effect size (η_p_^2^ 0.14) was imputed due to the lack of previous data assessing our main outcomes (sleep difficulty scores) in elite soccer players. With a power of 0.80 and α of 0.05, we calculated that a sample size of n = 63 was required to detect statistically significant effects.

### 2.2. Procedures

Players completed an online survey in the preseason/early season period of their respective 2023–2024 competitive seasons to determine their sleep behaviours and sleep quality. All male senior players completed the questionnaire between 4 and 79 days following the commencement of the preseason, all but one female senior player completed the questionnaire on day 36 following the commencement of the preseason (the exception completed it on day 51). Finally, all U21 male players completed the questionnaire between 3 and 8 days following the commencement of the preseason. Players were sent the survey via a link to their personal mobile phones or were given access via a club-owned tablet. All surveys were completed either at the training centre or at a training camp while a staff member was available to support their completion. All players other than 5 senior males completed the survey during the preseason period. Two of these five players were new signings who could not complete the survey during the preseason period; all five completed the survey within two months of the first EPL fixture. No players completed the survey on the day prior to a match, as the focus on these days was the next day’s competition, and full concentration and compliance were desired for questionnaire completion. All data was collected between July 1st and September 30th.

The survey was composed using electronic software (Qualtrics, Provo, UT, USA) and consisted of the following questions: (1) Please enter your name, (2) Please enter your age, and (3) Please select your team (Men’s first team, Women’s Team, Men’s Academy Team), followed by the questions that form the ASSQ and ASBQ. Players were asked to provide answers in relation to the prior 2 months, and, therefore, the data is representative of the off-season and preseason periods, and in some cases the end of the 2022–2023 season. For the 5 later completions, this also included some of the 2023–2024 competitive season. A Brazilian Portuguese translation was made available for non-English speaking players in the men’s senior squad; four players utilised this option. This translated version of the ASBQ was previously validated by Facundo et al. [48]. In the absence of an alternative language option for the ASSQ, Google Translate was used to generate a Brazilian Portuguese version for these 4 players. The scores from this version have not been validated.

### 2.3. Questionnaires

The ASSQ was developed to identify any clinical sleep issues that may require an intervention to improve sleep habits [49]. The questionnaire includes questions relating to sleep difficulties, sleep-disordered breathing, travel, and chronotype. The ASSQ was validated in a mixed-gender cohort against a sleep medicine physician’s clinical categorisation [49,50] and has acceptable test–retest reliability [51]. McDonald’s ω was 0.707, 0.501, and 0.549 for the male senior, female senior, and U21 male teams, respectively. For the items of the ASSQ, a numeric value was designated as per the validation [49]. The original questionnaire indicated that questions should be answered in reference to “the recent past”; to reduce ambiguity, we amended this to 2 months. Five questions (items 1, 3, 4, 5, and 6) were summed to give a sleep difficulty score (SDS). These questions are (1) During the recent past, how many hours of actual sleep did you get at night?; (2) How satisfied/dissatisfied are you with the quality of your sleep?; (3) During the recent past, how long does it usually take for you to fall asleep each night?; (4) How often do you have trouble staying asleep?; and (5) During the recent past, how often have you taken medicine to help you sleep (prescribed or over the counter)? Thresholds for the SDS are 0–4, None; 5–7, Mild; 8–10, Moderate; and 11–17, Severe.

The ASBQ was developed to evaluate individual sleep behaviours in athletes [50] and was used in this study to examine behavioural differences between squads. The ASBQ was validated against three previously validated sleep questionnaires and has acceptable test–retest reliability [50]. McDonald’s ω was 0.710, 0.740, and 0.624, for the male senior, female senior, and U21 male teams, respectively. The 18 items of the ASBQ were assessed using a 5-point Likert scale (Never = 1, Rarely = 2, Sometimes = 3, Frequently = 4, Always = 5). All responses were summed to give an ASBQ score between 18 and 90, with a higher global score equating to worse sleep behaviours. Thresholds are 36, Good; 37–41, Moderate; and >42, Poor.

### 2.4. Statistical Analysis

Statistical significance was set at *p* < 0.05 prior to analysis. For SDS and ASBQ scores, group differences were analysed with a one-way ANOVA (Welch’s Test). Scores were set as the dependent variables and team as the grouping variable. If a main effect was present, *post hoc* tests were used to ascertain where the differences were located, using the Games–Howell approach for multiple comparisons. Welch’s ANOVA and Games–Howell *post hoc* tests were used, as they do not assume equal variance [52], making these the most appropriate tests for uneven groups. The same analysis was used for responses to each item of the ASBQ. Although the data were ordinal, they were still analysed using parametric tests, as a moderate departure from normal distribution does not markedly affect the analysis [53]. Likewise, Likert scales are not affected by parametric and non-parametric analysis [54]. In addition, with a large sample size, all forms of non-normality have less effect on both power and significance level [53]; hence, parametric tests were deemed appropriate.

Hedge’s *g* effect sizes are presented for *post hoc* tests, with the following thresholds for small, medium, and large effects, respectively: 0.20, 0.50, and 0.80 [55]. Likewise, unadjusted confidence intervals (CIs) are presented for *post hoc* tests. Partial eta squared effect sizes (η_p_^2^) were added for main effects; 0.010–0.06 was considered a small effect, 0.06–0.14 a medium effect, and ≥0.14 a large effect [56].

Statistical analyses, including the calculation of McDoanld’s ω for reliability, were carried out in Jamovi (The Jamovi project, 2022). Jamovi (Version 2.4.7), excluding effect sizes and 95% confidence intervals, which were calculated using online calculators (Hedge’s G Calculator, 2021, retrieved from https://www.statology.org/hedges-g-calculator/ (accessed on October 2024); Means Difference Confidence Interval Calculator, retrieved from https://www.statskingdom.com/difference-confidence-interval-calculator.html (accessed on October 2024)). Confidence intervals were not adjusted for multiple comparisons.

## 3. Results

All 74 players gave consent and hence were deemed eligible and included in the analysis. All athletes completed all questions of the survey; there was no missing data.

### 3.1. Athlete Sleep Screening Questionnaire

There were significant differences between groups for the SDS (*p* = 0.003, η_p_^2^ = 0.29). Prevalence and qualitative inferences for the SDS are shown in Table 1. Male senior players had the lowest SDS (4.1 ± 1.7), followed by male U21 players (4.3 ± 1.2) and female senior players (5.9 ± 1.9). *Post hoc* analysis revealed a significant difference between male senior and female senior players (*p* = 0.003, *g* = 1.01, 95% CI, −2.90 to −0.76) and between female senior and male U21 players (*p* = 0.006, *g* = 1.00, 95% CI, 0.60 to 2.52) but not between male senior and male U21 players (*p* = 0.783, *g* = 0.19, 95% CI, −1.08 to 0.54).

### 3.2. Athlete Sleep Behaviour Questionnaire Scores

The number of players who scored >42 in the ASBQ, indicating poor sleep, was seven, nine, and one in the male senior, female senior, and U21 males, respectively. There were significant differences between groups for the global ASBQ scores (*p* < 0.001, η_p_^2^ = 0.24). Male U21 players scored the lowest on the ASBQ (33.6 ± 4.7), followed by male senior players (37.9 ± 6.5) and female senior players (40.6 ± 7.1). Post hoc analysis revealed a significant difference between male senior and male U21 players (*p* = 0.021, *g* = 0.77, 95% CI, 1.22 to 7.55) and between female senior and male U21 players (*p* < 0.001, *g* = 1.19, 95% CI, 3.46 to 10.66) but not between male seniors and female seniors (*p* = 0.372, *g* = 0.40, 95% CI, −6.66 to 1.30).

Athlete Sleep Behaviour Questionnaire responses are presented in Table 2.

For item 2 (stimulant use), *post hoc* tests revealed that there was a significant difference between male senior and male U21 players (*p* = 0.013, *g* = 0.82, 95% CI, 0.28 to 1.48) but not between male senior and female senior players (*p* = 0.594, *g* = 0.29, 95% CI, −0.37 to 1.08) or female senior and male U21 players (*p* = 0.257, *g* = 0.48, 95% CI, −0.14 to 1.20).

For item 3 (late night exercise), there was a significant difference between male senior and female senior players (*p* = 0.033, *g* = 0.75, 95% CI, 0.13 to 0.99) but not between male senior and male U21 players (*p* = 0.861, *g* = 0.14, 95% CI, −0.33 to 0.56) or female senior and male U21 players (*p* = 0.139, *g* = 0.56, 95% CI, −0.90 to 0.02).

For item 4 (late-night alcohol consumption), *post hoc* analysis revealed no significant differences between groups.

For item 6 (thirst at bedtime), there was a significant difference between male senior and female senior players (*p* = 0.008, *g* = 0.92, 95% CI, −1.15 to −0.25) but not between male senior and male U21 players (*p* = 0.535, g = 0.30, 95% CI, −0.77 to 0.24) or female senior and male U21 players (*p* = 0.234, *g* = 0.45, 95% CI, −0.09 to 0.96).

For item 9 (sport performance rumination), there was a significant difference between female senior and male U21 players (*p* < 0.001, *g* = 1.24, 95% CI, 0.55 to 1.54) but not between male senior and female senior players (*p* = 0.286, *g* = 0.43, 95% CI, −1.00 to 0.13) or between male senior and male U21 players (*p* = 0.072, *g* = 0.63, 95% CI, 0.07 to 1.16).

For item 10 (non-sport rumination), there was a significant difference between male senior and male U21 players (*p* = 0.020, *g* = 0.78, 95% CI, 0.21 to 1.25) and female senior and male U21 players (*p* < 0.001, *g* = 1.51, 95% CI, 0.74 to 1.71) but not between male senior and female senior (*p* = 0.242, *g* = 0.47, 95% CI, −1.10 to 0.11).

For item 12 (bathroom use), there was a significant difference between female senior and male U21 players (*p* = 0.043, *g* = 0.76, 95% CI, 0.15 to 1.42) but not between male senior and female senior (*p* = 0.080, *g* = 0.66, 95% CI, −1.35 to −0.06) or male senior and male U21 players (*p* = 0.941, *g* = 0.09, 95% CI, −0.39 to 0.54).

For item 14 (muscle twitching), there was a significant difference between female senior and male U21 players (*p* = 0.007, *g* = 1.03, 95% CI, 0.43 to 1.78) but not between male senior and female senior (*p* = 0.135, *g* = 0.59, 95% CI, −1.46 to 0.02) or male senior and male U21 players (*p* = 0.234, *g* = 0.46, 95% CI, −0.08 to 0.85).

For item 17 (sleeping in foreign environments), there was a significant difference between male senior and male U21 players (*p* = 0.045, *g *= 0.69, 95% CI, 0.01 to 1.13) but not between male senior and female senior players (*p* = 0.994, *g* = 0.03, 95% CI, −0.65 to 0.72) or female seniors and male U21 players (*p* = 0.101, *g* = 0.64, 95% CI, 0.03 to 1.21).

For item 18 (routine disrupted by travel), there was a significant difference between male senior and male U21 players (*p* = 0.010, *g* = 0.85, 95% CI, 0.25 to 1.21) but not between male senior and female senior players (*p* = 0.803, *g* = 0.18, 95% CI, −0.35 to 0.67) or female seniors and male U21 players (*p* = 0.060, *g* = 0.68, 95% CI, 0.08 to 1.06).

## 4. Discussion

The aim of this study was to use two validated questionnaires to establish and compare the sleep characteristics, difficulties, and behaviours of elite senior male, senior female, and U21 male soccer players during the preseason. The main finding was that senior female players reported greater sleep difficulty than their male counterparts. There were also several differences in sleep behaviours between squads; notably, female players reported greater pre-bed thirst, more frequent bathroom visits, and more night-time rumination, all of which may contribute to their greater sleep difficulty. None of the players reported severe difficulties with sleep; however, we have identified habits and behaviours that represent potential targets for interventions designed to enhance sleep quality.

Female players had a slightly higher SDS than senior male and U21 male players, but no players were categorised as having severe clinical sleep problems. Female players’ SDS categorises them as having mild sleep difficulty, which is in line with studies sampling the general population, where females reported worse sleep quality [57], and an increased predisposition to insomnia [58] than males. In athletes, the data is equivocal; some studies report no sex differences in sleep quality [25,59] but others have found significant differences in sleep quantity and quality, as measured by sleep efficiency and wake time [26,60] or subjective ratings [23]. However, these studies used different methods to measure sleep quality than the present study. In a previous study [61], female athletes also had a significantly higher SDS than male athletes (6.0 vs. 5.7). In contrast, [62] found no difference in SDS scores between males and females in national-grade Chinese athletes from a range of sports. The equivocal findings in the literature are likely due to different methods used to measure sleep difficulty, the timing of measurement (e.g., off-season vs. in-season), and differences in sport and athlete level [63]. In the current study, the higher SDS in females compared to males could be partly explained by different lifestyle demands [39] or different sleep habits, as indicated by the ASBQ responses (Table 2). Regarding hormonal factors, fluctuations in hormones may cause varying sleep difficulties throughout the menstrual cycle [28,29]. Lower subjective sleep quality is reported in the early follicular/menstrual and late luteal phases [28], and lower SE has been reported in the late luteal phase [29]. In the luteal phase, difficulties stem from increased progesterone, which is associated with higher body temperature, making it harder for women to fall asleep [64]. In the menstrual and early follicular phases, sleep could be negatively affected by pain or general discomfort [64]. As we did not include questions related to the menstrual cycle or contraception in this study, we can only speculate on whether they could have influenced sleep. Given that the surveys were answered in relation to the previous 2 months, any effects of different menstrual cycle phases could have been averaged out.

There are other possible reasons that could explain why female players reported a higher SDS. For example, female players reported waking to go to the bathroom more often than males. Although contrary to previous findings that women and men are equally likely to report going to the bathroom [65], this may have negatively influenced the SDS. Interestingly, female players also reported going to bed thirstier than male senior players (a large effect size); perhaps these differences are partly explained by females being more likely to report physical symptoms [66]. This also indicates possible differences in hydration levels between male and female players, warranting further research.

The U21 male players had the lowest ASBQ scores, showing significantly better behaviours than senior males and females (moderate to large effect sizes). The U21 global ASBQ scores in the present study (33.6) were better than those reported in varsity students aged ~20 years old (42.6) [67], and perhaps this is a reflection of the professionalism of the EPL Category 1 Academies. The difference between male U21 and senior players could be partly explained by fewer external influences; indeed, senior players’ sleep habits are more likely to be influenced by family commitments or young children, as well as media and sponsorship responsibilities. Alternatively, younger athletes have also been reported to avoid reporting sleep issues for fear of negatively influencing team selection [68], and this could have partly explained our findings. The major reasons for these differences should be explored by further research.

The questionnaire responses revealed a prevalence of further poor sleep habits. Across all players, 66% reported frequently or always using light-emitting technology (e.g., smartphones, computers, televisions) in the hour before bed. These findings concur with a study of elite track and field athletes, using the same survey, where the use of these light-emitting technologies was also prevalent [69]. It has been suggested that the use of electronic devices close to bedtime disrupts sleep [70]; however, the use of technology before bedtime is not unique to athletic populations, with 90% of the American general population [71] and young adult UK population [70] reporting the use of technology in the hour preceding bedtime.

Across all groups, 24% of players reported *sometimes*, *frequently*, or *always* taking naps over 2 h. Although longer naps may have occasional benefits, they can negatively influence night-time sleep via reduced sleep propensity [72]. As the questionnaire covered a period of the off-season, longer naps may have been more common. In addition, 16% reported worrying about sporting performance in bed, and 14% reported worrying about issues not related to sport. Interestingly, these were significantly greater in female senior players with especially large effect sizes compared to male U21 players. High levels of rumination and cognitive arousal can disrupt sleep [73] and have also been reported in previous studies with soccer players or other athletes [3,74]. Our findings on worrying about sporting performance in bed from the ASBQ (~2.0–~3.1; Table 2) are similar to those reported by Mason and colleagues [69] in elite male and female track and field athletes during habitual training or training camp (~2.3–~2.8). As no separate analysis on male and female athletes was performed in that study, it is unclear if they also found higher levels of worry in females as in the present study compared to U21 males. U21 male players also worried less than male and female senior players, possibly because they have less external and competition stress compared to senior players. These findings suggest that interventions designed to lower psychological arousal, such as mindfulness practices, could be employed by players reporting frequent worry and rumination [75,76]. Interventions may also be more valuable for female players, who reported significantly greater worry than male U21 players.

Senior males reported more frequent use of stimulants (15% reported frequent or always) than U21 male players (large effect size) and more late-night exercise activity (38% reported sometimes or more). These findings somewhat concur with previous studies; in a study of male English professional soccer clubs, 97% indicated providing caffeine to enhance performance, with 37% acknowledging use prior to training sessions [77]. However, their data was not collected with the ASBQ, limiting direct comparisons with the present study. U21 male players may be less likely to use dietary supplements and consume alcohol, which could partly explain why stimulant and alcohol use was recorded as lower in this cohort. The increased volume of late-night activity in male seniors may be partly explained by preseason training and match requirements, with some evening fixtures not finishing until ~21:30. Although late matches may negatively influence sleep [17], this is not a modifiable behaviour. However, the disruptive effects of late-night matches may be further compounded by stimulants, such as caffeine, which can disrupt sleep when taken even 6 h before bedtime [78]. Indeed, in professional rugby players, one study [79] showed that players who ingested caffeine before matches had worse sleep. Future research is required to determine which simulants are used and in what quantities.

This study has limitations to acknowledge. Firstly, our sample was drawn from a single elite soccer club in England and, therefore, the data may not be generalisable to soccer teams in other leagues. Secondly, sleep quality and quantity were not measured objectively; rather, we focused on sleep habits and behaviours, measured subjectively. Thus, we cannot associate the reported sleep behaviours with objective markers of sleep quality or quantity. Qualitative data may have offered greater insight into the sex differences, and interviews might be considered for future research to gain qualitative insights. A non-validated Brazilian Portuguese ASSQ translation was used for four players, and five of the male athletes completed their questionnaire in-season, both of which could have introduced bias into the findings. However, because this was only <10% of participants, it is unlikely to have significantly affected the findings. Furthermore, sub-analysis with the players using the Brazilian Portuguese translation removed had no influence on our main study outcomes. The internal consistency of the survey results for some teams was low (<0.70). As the ASBQ is based on gathering practical behavioural information (Table 2) and not how the responses relate to each other, this is unlikely to affect how the itemised behavioural responses would be interpreted; however, this could have influenced the findings for the ASSQ scores, and this should be considered when interpreting the findings. In addition, because of the timing of the survey completion during the preseason and early season, these results may not translate to other periods of the competitive season. The summer timing of completion provides different challenges to later in the season, especially the end of the season where fatigue has accumulated but performance pressure is perhaps highest. However, the preseason was selected to maximise player compliance and avoid distractions during competition. If feasible, future studies could include measures at other time points of the season for comparisons in a longitudinal design. Despite these limitations, this study was the first to investigate and compare sleep habits and behaviours across three different squads in the same EPL Club, all competing in the top leagues. This is also the first study to examine sleep behaviours in elite female soccer players, who are underrepresented in sports science research. The findings provide important information for sports medicine practitioners working in elite soccer that can be used to design sleep hygiene interventions.

## 5. Conclusions

In conclusion, we found that female players reported greater difficulty sleeping than senior male or male U21 soccer players, as measured subjectively with a validated survey. We also found that male senior players reported more travel disruption and use of stimulants, and female players reported more rumination and worry than male U21 players. Our data illustrates the diversity of behaviours exhibited by different cohorts of elite soccer squads and provides preliminary data that practitioners can use in the design of targeted intervention strategies to improve sleep quality in each specific cohort. From a practical perspective, our data suggest that female soccer players may need more support than male soccer players to enhance sleep quality. While further research is needed to confirm these findings in other soccer clubs and leagues, governing bodies and sports teams can utilise these findings to help them design practical strategies to support soccer players’ sleep quality.

## Figures and Tables

**Table 1:** Descriptive statistics of ASSQ (SDS score).

SDS Score Category	Male Senior	Female Senior	Male U21	All Players
None	73.1%	36.4%	50.0%	54.1%
Mild	19.2%	45.2%	50.0%	37.8%
Moderate	7.7%	18.2%	0.0%	8.1%
Severe	0.0%	0.0%	0.0%	0.0%

ASSQ, Athlete Sleep Screening Questionnaire; SDS, sleep difficulty score.

**Table 2:** ASBQ questions and scores for male senior, female senior, and U21 male soccer players.

Item	Question	Male Senior	Female Senior	Male U21	*p*-Value
**1**	I take afternoon naps lasting two or more hours	2.1 ± 1.1	1.6 ± 1.0	1.8 ± 1.3	0.181
**2**	I use stimulants when I train/compete	2.3 ± 1.2	2.0 ± 1.3	1.4 ± 0.9	0.016 *
**3**	I exercise (train or compete) late at night (after 7 p.m.)	2.2 ± 0.7	1.6 ± 0.7	2.1 ± 0.8	0.035 *
**4**	I consume alcohol within 4 h of going to bed	1.4 ± 0.7	1.3 ± 0.5	1.1 ± 0.3	0.035 *
**5**	I go to bed at different times each night (more than ± one hour variation)	2.2 ± 0.7	2.4 ± 0.6	2.2 ± 0.5	0.616
**6**	go to bed feeling thirsty	1.6 ± 0.8	2.3 ± 0.8	1.9 ± 1.0	0.011 *
**7**	I go to bed with sore muscles	2.4 ± 0.8	2.6 ± 0.7	2.4 ± 0.7	0.294
**8**	I use light-emitting technology in the hour leading up to bedtime (e.g., laptop, phone, television, video games)	3.5 ± 1.5	3.6 ± 1.5	4.0 ± 1.2	0.500
**9**	I think, plan and worry about my sporting performance when I am in bed	2.6 ± 1.1	3.1 ± 0.8	2.0 ± 0.8	<0.001 *
**10**	I think, plan and worry about issues not related to my sport when I am in bed	2.5 ± 1.1	3.0 ± 1.0	1.7 ± 0.7	<0.001 *
**11**	I use sleeping pills/tablets to help me sleep	1.4 ± 0.8	1.4 ± 0.9	1.1 ± 0.3	0.122
**12**	I wake to go to the bathroom more than once per night	1.9 ± 0.9	2.6 ± 1.3	1.8 ± 0.8	0.047 *
**13**	I wake myself and/or my bed partner with my snoring	1.2 ± 0.5	1.3 ± 0.8	1.2 ± 0.5	0.661
**14**	I wake myself and/or my bed partner with my muscle twitching	1.7 ± 1.0	2.4 ± 1.4	1.3 ± 0.6	0.005 *
**15**	I get up at different times each morning (more than ± one hour variation)	2.2 ± 0.6	2.3 ± 0.6	2.1 ± 0.7	0.612
**16**	At home, I sleep in a less than ideal environment (e.g., too light, too noisy, uncomfortable bed/pillow, too hot/cold)	1.4 ± 0.7	2.1 ± 1.1	1.5 ± 0.7	0.082
**17**	I sleep in foreign environments (e.g., hotel rooms)	2.8 ± 1.1	2.8 ± 1.2	2.2 ± 0.7	0.023 *
**18**	Travel gets in the way of building a consistent sleep-wake routine	2.6 ± 0.9	2.5 ± 0.9	1.9 ± 0.8	0.009 *

Data are mean ± standard deviation. Items are assessed using a 5-point Likert scale (Never = 1, Rarely = 2, Sometimes = 3, Frequently = 4, Always = 5. * indicates a significant difference between groups (*p* < 0.05). ASBQ, Athlete Sleep Behaviour Questionnaire.

## Data Availability

Due to the sensitive nature of the data, players did not give informed consent for the data to be shared beyond the research team.
